# Hospice Use Among Cancer Decedents in Alabama, 2002-2005

**Published:** 2009-09-15

**Authors:** Kathryn L Chapman, Todd M. Jenkins, Dorothy S. Harshbarger, Julie S. Townsend

**Affiliations:** Alabama Department of Public Health; Cincinnati Children’s Hospital Medical Center, Cincinnati, Ohio; Alabama Department of Public Health, Montgomery, Alabama; Centers for Disease Control and Prevention, Atlanta, Georgia

## Abstract

**Introduction:**

Most studies that describe hospice use among cancer patients use the Surveillance, Epidemiology, and End Results (SEER)-Medicare database, which has known limitations. We used vital records data to describe patterns of hospice use among cancer decedents in Alabama.

**Methods:**

To ascertain hospice use, we linked death certificates from 2002 through 2005 for people who died from cancer to listings of deaths reported by hospices. To evaluate accessibility of care, we calculated straight-line distances between decedent residence at death and the hospice providing care. We used these distances to estimate the reach of each hospice and identify the number of hospice nonusers residing in these areas.

**Results:**

During the study period, 52.0% of cancer decedents in Alabama received hospice care from 165 hospices. Nearly two-thirds of Alabama counties contain at least 1 hospice. Whites (53.6%) used hospice at a significantly higher rate than blacks (47.0%), but the rate of use was similar for women (53.2%) and men (51.0%). For people who were eligible for Medicare, 53.0% received hospice care. The median distance between decedent’s residence and the hospice providing care was 9.8 miles. This distance was slightly shorter for blacks than whites and roughly equal by sex.

**Conclusion:**

Alabamians use hospice at lower rates than observed elsewhere. Barriers to hospice care in Alabama must be identified and addressed.

## Introduction

The 1982 Medicare hospice benefit allowed beneficiaries with a life expectancy of 6 months or less to exchange curative care for comprehensive hospice care (1). Since then, the number of hospices providing care in the United States increased from approximately 1,500 in 1985 to 4,500 in 2006 (2). In 2006, an estimated 36% of all deaths in the United States occurred while the patient was under the care of a hospice program (3). Despite the widespread adoption of hospice services, an Institute of Medicine report concluded that a substantial number of people continue to experience needless distress at the end of life that might be alleviated by hospice care (4).

Historically, cancer patients have made up the largest proportion of hospice users, although this percentage has been declining (2). Since nearly half of all hospice users are cancer patients, hospice use among cancer patients has been described by using the Surveillance, Epidemiology, and End Results (SEER)-Medicare database (5-11). In 2007, 65% of Medicare recipients dying from cancer received hospice care (12), but few studies describe hospice use among cancer patients outside the SEER-Medicare population. A 2006 study used health maintenance organization (HMO) administrative data to describe hospice use among cancer patients (13). Although that investigation provided estimates for all cancer deaths (people aged 21 years and older), generalizability may have been limited because it was conducted in an HMO — a population that uses hospice services at substantially higher rates than does the general population (14).

To overcome deficiencies in previous studies, we used death certificate data and other administrative reports from the Alabama Department of Public Health (ADPH) Center for Health Statistics to describe and compare patterns of hospice use among cancer decedents of all ages in Alabama. Recognizing that some people are unaware of the services and support through end-of-life care and are unprepared for their own death or the death of a loved one, and that some health care professionals are not prepared to talk with family and patients about these issues, we developed the "End-of-Life Care" section of the Alabama Comprehensive Cancer Control Coalition (ACCCC) 2006-2010 Plan (15) to promote public awareness and educate health care professionals about these issues. By establishing a baseline metric for hospice use, we can evaluate the end-of-life care objectives outlined in the plan. To our knowledge, this is the first investigation to ascertain hospice use among cancer patients primarily on the basis of death certificate information.

## Methods

### Hospice use

We used death certificate and other administrative records to identify hospice use before death. We obtained death certificates from January 1, 2002, through December 31, 2005, for Alabama residents who died from cancer (International Statistical Classification of Diseases and Related Health Problems, 10th Revision [ICD-10] codes C00-C97) in Alabama (N = 37,864) from the Alabama Center for Health Statistics. By law, the physician in charge of care for the patient is responsible for providing the cause of death on the death certificate; if the person was not under the care of a physician, the coroner or medical examiner determines the cause of death (16).

To ascertain hospice use for each decedent, we manually matched death certificates to the hospice that administered care by using listings of deaths reported by hospices. To verify that a death certificate is filed for each deceased person in the state, Alabama law (17) requires every health care institution, including hospices, to provide a monthly listing of all deaths that occur under their care to the state registrar of vital records. We merged data from these monthly hospice-specific death reports to the corresponding death certificates, creating a new death file that included a hospice identifier. Since all hospice-reported deaths were matched to a death certificate, we could examine demographic and cause of death information by the specific hospice that provided care at the time of death and compare that information with information for decedents who did not receive hospice care.

### Geocoding

Alabama hospice facilities are primarily offices where business is conducted; 2 residential hospices with 10 beds each were included in the data. To visually assess use of hospice care, we geocoded all decedent and hospice facility addresses to the street level by using ArcView version 9.2 (Environmental Systems Research Institute, Inc, Redlands, California) and a Web-based geocoding application at www.batchgeocode.com. We geocoded a random sample of hospice addresses, using both ArcView and batchgeocode.com, to evaluate the validity of results using the online geocoding tool. We calculated distances between each method's geocoded location by using the Great Circle Method (18). This metric determines the shortest straight-line distance between 2 points (geocoded *x* and *y* coordinate values) on the earth's surface, accounting for the curvature of the earth. Addresses geocoded by the 2 methods differed in geographic position by an average of 0.29 miles (n = 100; 95% confidence interval, 0.08-0.51 miles; *P* = .01). Although the 2 methods produced significantly different results in terms of geocoded location, the observed difference was not considered to meaningfully affect results in this investigation. Previous findings in the literature have indicated that the positional accuracy of geocoded locations obtained with ArcView software was equivalent to those provided by commercial firms (19). Given the comparable level of accuracy with ArcView, we determined that the online tool at www.batchgeocode.com was a well-founded geocoding method for this investigation.

We geocoded decedent addresses by using a stepwise process. We first geocoded addresses with ArcView's StreetMapUSA reference data (2000 Topologically Integrated Geographic Encoding and Referencing system [TIGER] street data). We then geocoded addresses that were not matched in this first stage by using the online tool at www.batchgeocode.com. We geocoded records not matched by either method to their zip code centroid (center point of the zip codes). Most decedents (31,352 of 37,864, 83%) were geocoded in ArcView, and 17% were geocoded (6,437 of 37,864) with the Web-based application. Less than 1% of cancer deaths (73 of 37,864) were geocoded to their zip code of residence centroid. We were unable to geocode 2 death certificates that were completely missing address information.

We created maps that depicted county-level hospice use rates (by quartiles) in ArcView. We calculated distances between residence at death and the hospice that provided care by using the Great Circle Method (18). We also used this distance to estimate the reach of each hospice and capture the number of hospice nonusers residing in these areas.

### Decedent characteristics

We used the following fields from Alabama death certificates: year of death (2002, 2003, 2004, or 2005), race (white or black, which includes all nonwhite races, of which 99% are black), age at death (≤34, 35-44, 45-54, 55-64, 65-74, 75-84, or ≥85 years), marital status (never married, married, widowed, or divorced), ICD-10 underlying cause of cancer death (lung, C33-34; colorectal, C18-21; female breast, C50; prostate, C61; pancreas, C25; or all other cancers combined), and sex.

### Statistical analyses

We calculated crude rates of hospice use by each characteristic. Categorical variables were assessed by using χ^2^ tests, and continuous measures were examined with *t* tests. To compare our results with findings from SEER-Medicare–based investigations, we also calculated rates of hospice use among decedents aged 65 years or older at death. This research received approval from the University of Alabama at Birmingham institutional review board.

## Results

**Figure 1:**
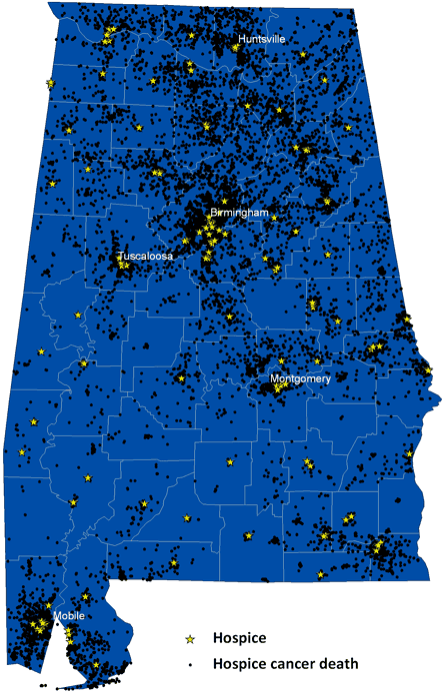
Location of hospices and cancer deaths under the care of a hospice, Alabama, 2002-2005.

**Figure 2:**
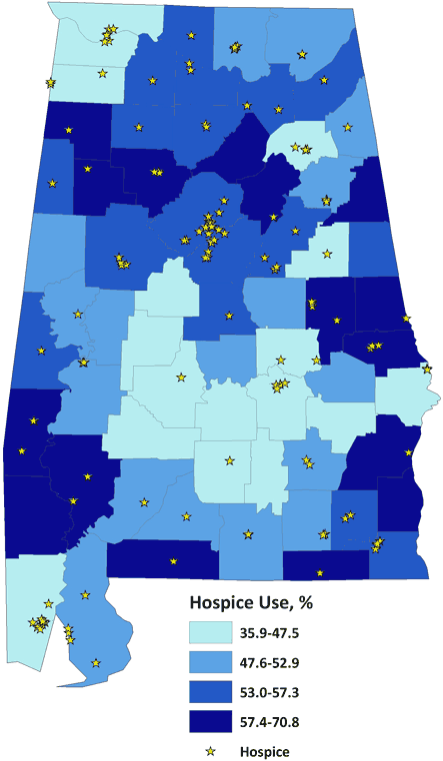
Hospice use among cancer decedents by county of death, Alabama, 2002-2005.

From 2002 through 2005, slightly more than half (52.0%) of Alabamians who died from cancer were receiving hospice care at the time of death (Table). In this period, 165 hospice entities provided care to these people, and 51of Alabama's 67 counties (76%) contained at least 1 hospice (Figure 1). Hospice use varied widely by county of residence, from a low of 35.9% in Butler County (75 of 209) to a high of 70.8% (461 of 651) in Lee County. Counties in south-central Alabama were in the lowest quartile of hospice use (35.9%-47.5%) (Figure 2). Several of these counties did not contain a hospice.

**Figure 3:**
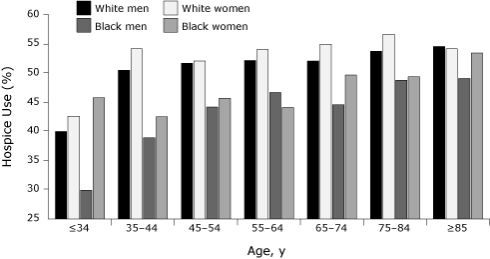
Hospice use among cancer decedents, by age at death, race, and sex, Alabama, 2002-2005.

Whites (53.6%) used hospice care at a significantly higher rate than did blacks (47.0%) (χ^2^ = 116.6, *df* = 1, *P* < .01), and the proportion of use for women (53.2%) was marginally larger than that for men (51.0%) (χ^2^ = 18.0, *df* = 1, *P* < .01). Slightly larger proportions of white women (54.7%) than white men (52.6%) (χ^2^ = 13.7, *df* = 1, *P* < .01) and of black women (48.2%) than black men (46.0%) (χ^2^ = 4.3, *df* = 1, *P* = .04) received hospice care. As expected, hospice usage rates significantly increased with age at death (*P* for trend <.01). White hospice users were comparable in age at death with black users (70.7 vs 68.9 years) (*t* = 7.14, *df* = 5,918, *P* < .01); female and male users were also similar in age at death (70.9 vs 69.7 years) (*t* = 6.25, *df* = 19,700, *P* < .01). Age-specific hospice use varied by race and sex (Figure 3). White women, followed by white men, had the highest rates of use across most age categories. Black men had the lowest rates of use for most age groups.

**Table:** 

**Race and Sex**	**Age at Death, y**						
	**≤34**	**35-44**	**45-54**	**55-64**	**65-74**	**75-84**	**≥85**
White men	40	50	52	52	52	54	54
White women	42	54	52	54	55	57	54
Black men	30	39	44	47	44	49	49
Black women	46	42	46	44	50	49	53

Slightly less than 70% of users were aged 65 years or older at death, and more black than white hospice users were younger than 65 at death (36.5% vs 29.5%). To compare these figures with results derived from SEER-Medicare data, the rate of hospice use was calculated for those aged 65 years or older at death. Of Medicare-eligible cancer decedents in Alabama, 53.0% received hospice care from 2002 through 2005.

**Figure 4:**
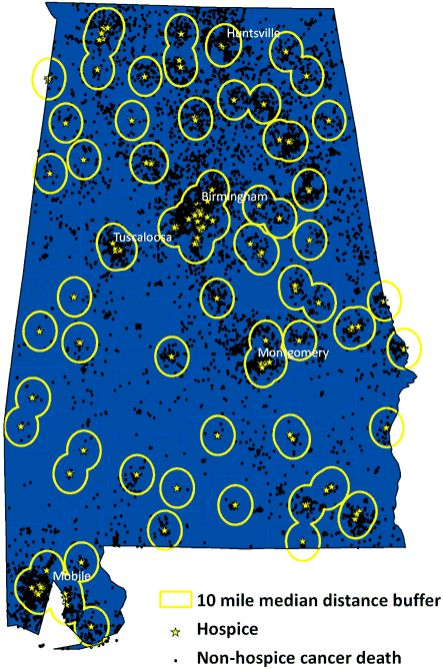
Location of hospices and cancer deaths among people who did not receive hospice care, Alabama, 2002-2005. Circles show the 10-mile radius around each hospice, which was the median distance between hospice and residence of hospice users; 60% of hospice nonusers would have been captured in this radius.

Overall, the median distance between decedent's residence and hospice location was 9.8 miles. This distance was shorter for blacks than whites (6.6 vs 10.6 miles) and roughly equal by sex. Among decedents who did not receive hospice care, 60% lived within 10 miles of a hospice, the median distance among users (Figure 4); 77% of nonusers lived within a 20-mile radius. Results did not vary by race or sex. Among hospice nonusers aged 65 or older at death, 64.2% lived within 10 miles and 77.2% were within 20 miles of a hospice.

## Discussion

In Alabama from 2002 through 2005, hospice use at the time of death for cancer patients was 52.0%, which is well below figures reported for other locations. From 1996 to 2001, 65.4% of HMO enrollees in northern California who died from cancers of the lung, colon-rectum, breast, or prostate received hospice care (13). In contrast, only 52% to 55% of comparable cancer deaths in Alabama (2002-2005) were among people who were under hospice care at death. This discrepancy may be partially explained by the fact that HMO enrollees have significantly higher rates of hospice use (14). However, given the difference in time between these studies, one would expect the difference in rates to be smaller, since hospice use, in general, has increased over time. This difference may reflect lower levels of hospice use in Alabama than in other parts of the United States.

Although most publications describing patterns of hospice use among cancer patients have relied on the SEER-Medicare database (5-11), this study was not limited by age or payer source. Slightly more than 30% of hospice users who died of cancer in Alabama were younger than age 65 at death, although this figure increased to 36.5% among blacks. Given these findings, investigations derived from the SEER-Medicare database may exclude a substantial portion of younger hospice users.

To compare results from this study with SEER-Medicare–based investigations, rates of use among those aged 65 years or older at death were calculated separately. During the 4-year study period, 53.0% of Medicare-eligible cancer decedents in Alabama received hospice care. A previous study reported that 65% of Medicare recipients who died from cancer in 2002 received hospice services (12). By comparison, hospice usage among cancer decedents aged 65 years or older in Alabama in 2002 was 52.2%. The discrepancy in estimates likely results from a combination of factors. Although the Medicare-eligible population was restricted to those aged 65 years or older, approximately 3% of eligible Americans are not enrolled in Medicare (20). Additionally, analyses using SEER-Medicare data can identify live hospice discharges (12), whereas our method prevented us from doing so. Live hospice discharge estimates range from 6% of all hospice users to 15.5% of Medicare recipients (21,22). Some investigations have also shown that people with a cancer diagnosis are significantly less likely to be discharged alive compared with those with diagnoses other than cancer (21), but other studies have found no such association (22).

Hospices in Alabama are regulated by the State Board of Health through the Division of Health Provider Services in the ADPH, with no certificate of need requirement. Currently, there is a moratorium on licensing new hospices. In accordance with Act 2006-617 of the 2006 Alabama Legislature (23), Alabama can issue a new hospice license only if an applicant has met specific requirements and if the application was filed by July 7, 2007, or the ADPH has inspected all licensed hospices in the preceding 12 months. Therefore, applications for new hospice licenses will not be accepted until the ADPH inspection process is current (24). This moratorium on new hospice licensing raised concerns for the Survivorship Workgroup associated with ACCCC. However, results of the analysis of catchment areas for each hospice found that 60% of hospice nonusers lived within 10 miles of a facility (the median distance among users), and 77% lived within 20 miles, which implies that distance is not a barrier to hospice care for most hospice nonusers in Alabama.

Strengths of this investigation primarily relate to the data sources used to ascertain hospice care. First, this study analyzed nearly 38,000 cancer deaths. The novel technique to determine hospice use is also an asset because this study was not restricted by age or payment method, since it was population-based. This method for determining hospice use also has benefits over studies that use death certificates alone. Many states are changing their death certificates to follow recommendations (25) presented for the 2003 US Standard Certificate of Death (26) that called for adding a box under "place of death" for "hospice facility" to distinguish those deaths from deaths that occurred in a hospital, nursing home or long-term care facility, decedent's home, or other location. Instructions for completing this new category state that "hospice facility refers to a licensed institution providing hospice care (eg, palliative and supportive care for the dying), not to hospice care that might be provided in a number of different settings, including a patient's home" (27). Thus, states that use this new version of the question would be able to examine characteristics of people who die in a hospice facility; however, they still would not be able to study deaths among all people under hospice care. By 2008, approximately half of the states had adopted this update (26).

This study has several limitations. Because this study measured hospice use at death, people who were discharged alive from a hospice facility were potentially misclassified as nonusers. Such misclassifications could result in underestimates of the true usage rates. These results may not be generalizable outside of Alabama, although rates of use in Alabama displayed many of the same patterns observed nationwide, albeit at a lower rate of use. Finally, positional accuracy of geocoding is lower for rural addresses (19), so distances calculated between residences and hospices in rural areas are likely to have a higher degree of error than in nonrural areas.

Our study provides valuable baseline data for the "End-of-Life Care" section in the ACCCC plan and reveals racial, geographic, and other disparities in hospice care use in Alabama. To increase awareness of hospice care, the ACCCC has taken steps to disseminate these findings. Using maps to visualize the varying patterns of use helps the ACCCC concentrate educational messages about hospice services in the areas of most need. In conjunction with the study results, the Alabama Hospice Organization has garnered wide support for a certificate of need process to replace the moratorium on new hospices. That hospice use in Alabama is somewhat lower than that observed nationally is a concern of the ACCCC. It recommends conducting additional studies to try to determine barriers that might prevent hospice use and determine whether family members have the appropriate education about the benefits that hospice care can provide to support the family as well as the patient. Such investigations are under way.

## Figures and Tables

**Table.** Cancer Deaths and Hospice Use, by Selected Characteristics, Alabama, 2002-2005

**Characteristic**	No. of Deaths	% Hospice Use (95% Confidence Interval)^a^
**Total**	37,864	52.0 (51.5-52.5)
**Year**		
2002	9,361	52.2 (51.2-53.2)
2003	9,482	53.0 (52.0-54.0)
2004	9,447	52.5 (51.5-53.5)
2005	9,574	50.5 (49.5-51.5)
**Race**		
White	29,107	53.6 (53.0-54.1)
Black^b^	8,757	47.0 (45.9-48.0)
**Sex**		
Male	20,501	51.0 (50.3-51.7)
Female	17,363	53.2 (52.5-54.0)
**Age, y**		
≤34	431	39.7 (35.1-44.3)
35-44	1,076	48.5 (45.5-51.5)
45-54	3,590	49.5 (47.9-51.1)
55-64	7,103	51.2 (50.0-52.3)
65-74	10,203	51.9 (50.9-52.8)
75-84	10,589	53.8 (52.9-54.8)
≥85	4,872	53.6 (52.3-55.1)
**Cancer site**		
Lung	11,888	52.5 (51.6-53.4)
Colorectal	3,470	53.2 (51.6-54.9)
Female breast	2,665	52.5 (50.6-54.4)
Prostate	2,123	54.7 (52.6-56.8)
Pancreas	1,932	59.3 (57.1-61.5)
Other	15,786	50.1 (49.3-50.9)

a Hospice reported death.

b Black race includes all nonwhite races, of which 99% are black.

## References

[1] Virnig BA, Moscovice IS, Durham SB, Casey MM (2004). Do rural elders have limited access to Medicare hospice services?. J Am Geriatr Soc.

[2] Growth in US hospice programs.

[3] (2007). NHPCO's facts and figures: Hospice care in America.

[4] Field MJ, Cassel CK (1997). Approaching death: improving care at the end of life.

[5] Ngo-Metzger Q, Phillips RS, McCarthy EP (2008). Ethnic disparities in hospice use among Asian Americans and Pacific Islander patients dying with cancer. J Am Geriatr Soc.

[6] Haas JS, Earle CC, Orav JE, Brawarsky P, Neville BA, Acevedo-Garcia D (2007). Lower use of hospice by cancer patients who live in minority versus white areas. J Gen Intern Med.

[7] Locher JL, Kilgore ML, Morrisey MA, Ritchie CS (2006). Patterns and predictors of home health and hospice use by older adults with cancer. J Am Geriatr Soc.

[8] Lackan NA, Ostir GV, Freeman JL, Kuo YF, Zhang DD, Goodwin JS (2004). Hospice use by Hispanic and non-Hispanic white cancer decedents. Health Serv Res.

[9] Lackan NA, Ostir GV, Freeman JL, Mahnken JD, Goodwin JS (2004). Decreasing variation in the use of hospice among older adults with breast, colorectal, lung and prostate cancer. Med Care.

[10] McCarthy EP, Burns RB, Ngo-Metzger Q, Davis RB, Phillips RS (2003). Hospice use among Medicare managed care and fee-for-service patients dying with cancer. JAMA.

[11] McCarthy EP, Burns RB, Davis RB, Phillips RS (2003). Barriers to hospice care among older patients dying with lung and colorectal cancer.. J Clin Oncol.

[12] Connor SR, Elwert F, Spence C, Chrisakis NA (2007). Geographic variation in hospice use in the United States in 2002. J Pain Symptom Manage.

[13] Keating NL, Herrington LJ, Zaslavsky AM, Liu L, Ayanian JZ (2006). Variations in hospice use among cancer patients. J Natl Cancer Inst.

[14] Virnig BA, Kind S, McBean M, Fisher ES (2000). Geographic variation in hospice use prior to death. J Am Geriatr Soc.

[15] (2005). Alabama Comprehensive Cancer Control Coalition 2006-2010 plan.

[16] Code of Alabama, 1975, section 22-9A-14.

[17] Code of Alabama, 1975, section 22-9A-24.

[18] Hamilton L Linking SAS analytics, US Census Gazetteer data, and ArcView geocoding software for Medicare fraud screening.

[19] Ward MH, Nuckols JR, Giglierano J, Bonner MR, Wolter C, Airola M (2005). Positional accuracy of 2 methods of geocoding. Epidemiology.

[20] O'Sullivan J, Lee JS, Yang B (1996). Medicare: the role of supplemental health insurance.

[21] Kutner JS, Blake M, Meyer SA (2002). Predictors of live hospice discharge: data from the National Home and Hospice Care Survey (NHHCS). Am J Hosp Palliat Care.

[22] Taylor DH, Steinhauser K, Tulsky JA, Rattliff J, Van Houtven (2008). Characterizing hospice discharge patterns in a nationally representative sample of the elderly, 1993-2000. Am J Hosp Palliat Care.

[23] Summaries of general laws enacted and constitutional amendments proposed by the legislature of Alabama at the regular session, January 10 to April 17, 2006.

[24] Alabama Department of Public Health. Notice new hospice applications.

[25] National Center for Health Statistics. Report of the Panel to Evaluate the US Standard Certificates, April 2000, addenda November 2001.

[26] National Center for Health Statistics. 2003 US standard certificate.

[27] (2004). Funeral directors' handbook on death registration and fetal death reporting, 2003 revision.

